# MicroRNA expression within neuronal-derived small extracellular vesicles in frontotemporal degeneration

**DOI:** 10.1097/MD.0000000000030854

**Published:** 2022-10-07

**Authors:** Jonathan Pounders, Emily J. Hill, Destiny Hooper, Xiang Zhang, Jacek Biesiada, Damaris Kuhnell, Hannah L. Greenland, Leyla Esfandiari, Emerlee Timmerman, Forrest Foster, Chenran Wang, Kyle B. Walsh, Rhonna Shatz, Daniel Woo, Mario Medvedovic, Scott Langevin, Russell P. Sawyer

**Affiliations:** a University of Cincinnati College of Medicine, Department of Neurology and Rehabilitation Medicine, Cincinnati, OH, USA; b University of Cincinnati College of Medicine, Department of Environmental and Public Health Sciences, Cincinnati, OH, USA; c University of Cincinnati, Department of Electrical Engineering and Computer Science, Cincinnati, OH, USA; d University of Cincinnati, Department of Biomedical Engineering, Cincinnati, OH, USA; e University of Cincinnati College of Medicine, Department of Cancer Biology, Cincinnati, OH, USA; f University of Cincinnati College of Medicine, Department of Emergency Medicine, Cincinnati, OH, USA.

**Keywords:** Alzheimer’s disease, exosomes, frontotemporal dementia, microRNA

## Abstract

MicroRNAs (miRNAs) are small non-coding RNA that are powerful regulators of gene expression and can affect the expression of hundreds of genes. miRNAs can be packed in small extracellular vesicles (SEV) and released into the extracellular space by neurons and microglia to act locally as well as pass through the blood-brain barrier and act systemically. We sought to understand the differences in neuronal SEV miRNA expression between frontotemporal dementia (FTD), Alzheimer’s disease (AD), and healthy aging. Plasma was obtained from FTD, AD, and healthy aging participants that were matched based on age, sex, and race/ethnicity. Additionally, a subset of participants also provided paired cerebrospinal fluid samples to compare neuronal SEV miRNAs in plasma and cerebrospinal fluid. Neuronal SEV were isolated using differential ultracentrifugation and antibody conjugated Dynabeads® for the neuronal surface marker, L1CAM. RNA sequencing was performed. 12 FTD, 11 with AD, and 10 healthy aging participants were enrolled in the study. In FTD, SEV miRNA-181c was downregulated compared to healthy controls. In AD, miRNA-122 and miRNA-3591 were downregulated compared to those in healthy controls and FTD. Using an FDR <0.2, only miRNA-21-5p was found to have increased expression in the cerebrospinal fluid compared to plasma in a group of AD and FTD participants. SEV miRNA-181c is significantly downregulated in FTD compared to healthy controls and may mediate its effects through microglial-directed neuroinflammation and interaction with TAR DNA-binding protein 43 (TDP-43) based on pathway analysis. Additionally, the FOXO and Hippo pathways may be important mediators of FTD, based on pathway analysis. Lastly, because only one SEV miRNA was differentially expressed between the plasma and cerebrospinal fluid in paired samples, plasma represents an appropriate biofluid for studying neuronal SEV miRNA.

## 1. Introduction

Frontotemporal dementia (FTD) is the most common cause of dementia before the age of 60 and the third most common neurodegenerative disease in any age group.^[1]^ While FTD is pathologically characterized by early atrophy of the frontal and temporal lobes, as well as abnormal protein aggregates within neurons, the pathophysiological mechanisms of FTD remain unknown. MicroRNAs (miRNA) are non-protein-coding RNA molecules that act as powerful regulators of post-transcriptional gene expression. A single miRNA molecule can regulate hundreds of genes.^[2]^ In humans, 2654 mature miRNA have been reported,^[3]^ many from within the nervous system, implicating them in a variety of neurodegenerative diseases including Parkinson’s disease, Huntington’s disease, amyotrophic lateral sclerosis (ALS), Alzheimer’s disease (AD), and FTD.^[4–7]^ In inherited forms of FTD caused by autosomal dominant mutations in the *C9orf72* or *FUS* genes, impaired biosynthesis of miRNA and reduced miRNA regulatory activity within cortical neurons have been identified.^[7–10]^ Since a single miRNA can regulate gene expression of hundreds of genes through post-transcriptional silencing of target mRNA, a multitude of complex disease pathways may be impacted by alterations in miRNA expression.^[2]^ Additionally, miRNA can also be transferred between neurons and other cells via small extracellular vesicles (SEV). SEV are tiny, 30 to 150 nanometer, membrane-encapsulated vesicles that are thought to mediate cell-to-cell communication through the horizontal transfer of genetic material and alterations in gene expression within the recipient cell.^[11]^ SEV are released by virtually all mammalian cells and are identifiable by surface markers specific to their origins.^[12]^ Within the central nervous system, SEVs secreted by neurons can pass through the blood-brain barrier into the bloodstream and cerebralspinal fluid (CSF), providing a unique opportunity to observe molecular changes within the central nervous system of patients with neurodegenerative diseases, without biopsy or autopsy.^[13,14]^ We sought to examine whether SEV miRNA profiles within neuron specific SEVs isolated from plasma differed among FTD, AD, and healthy aging controls.

## 2. Materials and Methods

### 2.1. Standard protocol approvals, registrations, and patient consents

The study protocol was approved by the University of Cincinnati Institutional Review Board. Informed consent was obtained from all participants. Consent was provided by a guardian/power-of-attorney for FTD and AD participants when necessary. Enrollment was completed between September 2020 and August 2021 and was based on patient consent during the study period.

### 2.2. Participants

Participants with FTD were diagnosed with probable or definite FTD based on the Rascovsky criteria for behavioral variant FTD or Gorno-Tempini criteria for primary progressive aphasia (PPA).^[15,16]^ Participants with AD, based on the Clinical Dementia Rating scale sum of boxes (CDR SOB) ≥1, A-beta 42 to Total-Tau Index ≤1.0, and hyperphosphorylated tau ≥61 pg/mL on CSF testing through Athena Diagnostics, were included. Participants were excluded if they had a concurrent neurodegenerative disorder (AD + FTD, Parkinson’s disease, or Lewy body disease) based on the diagnostic review of two behavioral neurologists at our center (RPS, RSS). Healthy controls were directly matched to subjects with FTD by age (±5 yr), race/ethnicity (by self-identification), and sex. Healthy controls were excluded if they or their informants reported cognitive decline in the previous year, and there was no evidence from a screening visit suggesting a neurodegenerative disorder (including neuropsychological assessment or CSF analysis with Alzheimer’s biomarkers) or clinically significant neurologic disorder as determined by the principal investigator. Healthy controls with a family history (3 degrees) of autosomal dominant neurodegenerative or neuropsychiatric diseases and individuals with a known mutation causing neurodegenerative disease were excluded. Participants with FTD underwent commercial genetic testing through PreventionGenetics, LLC or Variantyx Inc. to evaluate the presence of pathogenic variants associated with FTD, including *C9orf72* repeat expansions, *GRN*, and *MAPT*. This test was used to categorize but not exclude FTD participants.

### 2.3. SEV isolation and characterization

Plasma SEV were isolated using a previously described differential ultracentrifugation protocol^[17]^ that was validated in accordance with the minimal experimental guidelines established by the International Society for Extracellular Vesicles (ISEV).^[18]^ Briefly, plasma (3 mL) was diluted with an equal volume of PBS and centrifuged for 30 minutes at 2000 × *g* at 4°C. The supernatant was transferred to a new tube and centrifuged for 45 minutes at 12,000 × *g* and 4°C. The supernatant was collected; PBS was added to bring the total volume to 20 mL, which was gently pipetted on top of 4 mL Tris/sucrose/D_2_O solution (30% sucrose cushion) and centrifuged for 75 minutes at 100,000 × *g* at 4°C with an Optima L-100K ultracentrifuge using a 70Ti fixed-angle rotor. A 5 mL syringe fitted with an 18-G needle was used to collect approximately 3.5 mL of the sucrose cushion from the side of the ultracentrifuge tube. The aspirate was then transferred to a fresh ultracentrifuge tube, diluted to 60 mL with PBS, and centrifuged for 70 minutes at 100,000 × *g* and 4°C using a 45Ti fixed-angle rotor. The SEV pellet was resuspended in 100 μL of PBS and stored at −80°C for downstream analysis.

To reduce sample heterogeneity and enrich for neuronal-derived SEV, magnetic bead-based positive selection for each plasma SEV isolate^[19]^ using DSB-X biotin-labeled L1CAM antibody-conjugated Dynabeads^®^ Flow Comp Flexi (Invitrogen)^[20]^ was applied. The L1CAM antibody was DSB-X biotin-labeled using the DSB-X Biotin Protein Labeling Kit (Invitrogen). The L1CAM-positive (L1CAM[+]) SEV isolates were subsequently incubated with release buffer to decouple SEV from Dynabeads, after which the sample was placed in the magnet and the supernatant containing decoupled L1CAM (+) sEV.

The particle size distribution and concentration were subsequently determined for each L1CAM (+) SEV isolate via nanoparticle tracking analysis (NTA) using a NanoSight NS300 instrument (Malvern, Worcestershire, UK).

### 2.4. miRNA sequencing

miRNAs were sequenced by Genomics, Epigenomics, and Sequencing Core (GES Core) at the University of Cincinnati, using patented miRNA sequencing technology. Briefly, the NEBNext small RNA sample library preparation kit (New England BioLabs) used ~5 ng of total RNA as the input. The size selection of the miRNA library was performed using a novel approach optimized for low RNA input and conditions from SEV isolates (US Patent 62/266,902). First, a custom-made ladder was mixed with the pre-purified library as in-lane marks for precise cutting of the 135 to 146 bp range. Co-purification of the ladder increased library recovery and enabled the use of agarose gel, which simplified library purification. Second, optionally equal volumes of pre-purified libraries were pooled and mixed with our custom-made ladder, followed by gel purification and sequencing. Based on the read alignment from the test run, the volume of each pre-purified library was adjusted to generate the desired number of miRNA reads for each sample in subsequent sequencing. Libraries were clustered onto a flow cell at a concentration of 10 pM using a TruSeq SR Cluster Kit v3 (Illumina) and sequenced for 50 cycles using a TruSeq SBS kit on an Illumina NextSeq 550 platform.

### 2.5. Bioinformatics

Sequence reads were pre-processed to remove adapters and low-quality reads, such as reads containing more than 25 bases, reads containing more than four undefined reads (N), short reads (<16 bases), and long reads (>30 bases). Reads with lengths between 16 and 30 bases were aligned to the reference human genome (GRCh38) using the Bowtie aligner.^[21]^ The reads aligned to each known mature miRNA were counted using Bioconductor packages for next-generation sequencing data analysis^[22]^ based on miRNA definitions in the miRBase database version 22.^[23]^ Statistical analysis to detect differentially expressed miRNAs was performed based on the negative binomial model of read count as implemented in the edgeR^[24]^ R package. Statistical comparisons were adjusted for the false discovery rate (FDR).^[25]^ Differential expression was considered significant when the FDR-adjusted *P* value (q value) ≤ was .2. The mechanistic interpretation of changes in miRNA levels was facilitated by pathway analysis of predicted and validated targets^[26–28]^ using the specialized DIANA mirPath v.3.^[29–31]^ Enrichment *p* values were considered significant when q < 0.2.

### 2.6. Data availability statement

The raw data were generated at the University of Cincinnati. The data supporting the findings of this study are available from the corresponding author upon request.

## 3. Results

A description of the demographic and clinical characteristics of study participants is provided in Table 1. A significant difference was observed in the mean CDR SOB, with the FTD group having a higher mean CDR SOB score than the AD group (*P* = .04), indicating a more severe stage of neurodegenerative disease. Because healthy controls lacked cognitive impairment, their CDR composite and CDR SOB scores were both zero.

**Table 1:** Demographics.

	FTD	AD	HC
# Participants	12	11	10
Mean Age (std)	70.3 (7.33)	70.1 (7.29)	69.9 (6.82)
% Female	33	36	40
CDR Composite = 0.5	4	8	0
CDR Composite = 1	6	3	0
CDR Composite = 2	2	0	0
Mean CDR SOB	5.42 (3.26)	2.95 (1.98)	0

AD = Alzheimer’s disease, CDR = clinical dementia rating scale, FTD = frontotemporal degeneration, HC = healthy control, SOB = sum of boxes.

Twenty-three unique miRNA transcripts were identified in L1CAM (+) plasma SEV isolates. miRNAs are listed in Supplementary Table 1, http://links.lww.com/MD/H510. Compared to healthy controls, FTD participants had decreased expression of *miR-181c* (fold change = 0.15, log^2^ fold change = −2.72, q = 0.08). Compared to participants with AD, participants with FTD had higher expression of *miR-122* (fold change = 9.66, log^2^ fold change = 3.27, q = 0.003) and *miR-3591* (fold change = 9.66, log^2^ fold change = 3.27, q = 0.003). In AD participants, there was decreased expression of miRNA-122 (fold change = 0.21, log^2^ fold change = −2.27, q = 0.02) and miRNA-3591 (fold change = 0.21, log^2^ fold change = −2.27, q = 0.02) compared to that in healthy controls.

miRNAs with an FDR < 0.2 were evaluated using the KEGG pathway analysis package.^[27]^ 34 Cellular pathways with up to 111 genes implicated in their expression in FTD (Supplementary Table S2, http://links.lww.com/MD/H511) and 50 pathways with up to 149 genes implicated in their expression in AD (Supplementary Table S3, http://links.lww.com/MD/H512) compared to healthy controls.

Participants with FTD were stratified by disease severity (CDR) composite score, and expression of L1CAM (+) SEV-miRNA in CDR > 1 versus CDR = 1, CDR = 1 versus CDR = 0.5, and CDR > 1 versus CDR 0.5 severity groups were compared. There were no statistically significant differences in the expression of composite CDR > 1 or CDR = 0.5. Table 2 presents the results of this analysis are listed in Table 2. Then AD Participants with composite CDR = 1 versus CDR 0.5 severity groups were compared, though only three AD participants had composite CDR = 1. Three neuronal SEV miRNA were lower in AD participants with composite CDR = 1 compared to CDR = 0.5, see Table 3.

**Table 2:** Differences in L1CAM (+) SEV microRNA across FTD disease severity.

	log^2^ fold change	*p* value	Adjusted *p* value
CDR = 2 vs CDR = 1			
miRNA-184	3.74	.002	.08
miRNA-3168	2.88	.01	.19
Let-7i	-3.72	.03	.32
CDR = 1 vs CDR = 0.5			
miRNA-122	3.91	.007	.15
miRNA-3591	3.91	.009	.15
miRNA-10	4.42	.03	.24
miRNA-26	2.28	.03	.24
miRNA-203	−2.09	.04	.24
miRNA-3545	−2.09	.04	.24
Let-7i	1.81	.05	.24

CDR = clinical dementia rating scale composite score, miRNA = microRNA.

**Table 3:** Differences in L1CAM (+) SEV microRNA across AD severity.

	log^2^ fold change	*P* value	Adjusted *p* value
CDR = 1 vs CDR = 0.5			
miRNA-22	−3.65	.06	.88
miRNA-92	−3.02	.08	.88
miRNA-222	−2.34	.18	.88

CDR = clinical dementia rating scale composite score, miRNA = microRNA.

Finally, we performed an intra-subject comparison of the expression of L1CAM (+) SEV-miRNA isolated from plasma versus cerebrospinal fluid, where possible. Paired plasma-cerebrospinal fluid samples were available for three participants with AD and one participant with FTD. Using an FDR of <0.2, only miRNA-21-5p was found to have increased expression in the cerebrospinal fluid compared to the plasma (fold change = 4.48, log^2^ fold change = 2.16, q = 0.10). See Supplementary Table S4, http://links.lww.com/MD/H513 for a complete list of SEV miRNAs observed in the cerebrospinal fluid and plasma of the participants.

## 4. Discussion

In FTD, SEV miRNA-181c was downregulated compared to healthy controls. In AD, miRNA-122 and miRNA-3591 were downregulated compared to those in healthy controls and FTD. MicroRNA-181c is expressed primarily in the brain.^[32]^ Within the neuron, reduced miRNA-181c has been associated with dendritic branching, dendrite spine density, and reduced synaptogenesis.^[33,34]^ MicroRNA-181c has also been shown to interact with TAR DNA-binding protein 43 (TDP-43), one of the major cytoplasmic inclusions in FTD.^[35]^ Reduced production of microRNA-181c has been shown to correlate with translocation of TDP-43 from the nucleus to the cytoplasm.^[35]^ Authors suggested that TDP-43 disrupts a negative feedback network between itself and miRNA-181c.^[35]^ MicroRNA-181c has also been shown to regulate neuroinflammation by reducing microglial activation, promote microglial apoptosis,^[36]^ and reduce microglial expression of proinflammatory cytokines.^[37]^ Suggesting our findings of reduced SEV miRNA-181c may facilitate a proinflammatory state in the brain through increased release of microglial cytokines. Additionally, miRNA-181c has been shown to modulate inflammation under normal and autoimmune conditions in the central nervous system by reducing CD4 T-cell activation,^[38]^ as well as the subsequent production of proinflammatory cytokines IL-7 and IL-17,^[39]^ while promoting the differentiation of anti-inflammatory T helper cells. Thus, there is ample evidence implementing miRNA-181c is a key mediator of neuroinflammation, as well as of the peripheral immune response. This relationship and its mechanisms need to be explored further in the context of FTD and neurodegenerative diseases.

The expression of miRNA-122 and miRNA-3591 was relatively reduced in patients with AD compared to that in healthy controls and FTD patients. While most of the literature exploring the in vivo role of miRNA-122 has been dedicated to liver disease and hepatocellular carcinoma, Gu et al found that overexpression of miRNA-122 was seen in AD mouse models and could be related to signaling mechanisms in amyloid-β-induced neuronal apoptosis.^[40]^

In addition to serving as possible biomarkers for both FTD and AD, differentially expressed miRNAs revealed downstream targets that could be associated with neurodegeneration. Both the FTD-control and AD-control comparisons showed differential expression of miRNAs and multiple affected cellular pathways (Supplementary Tables S2 http://links.lww.com/MD/H511 and S3 http://links.lww.com/MD/H512), including the FOXO and Hippo signaling pathways. The Hippo signaling pathway is an apoptosis regulator that is essential for normal organismal development and physiological homeostasis; however, its diminished activity can lead to excessive cellular proliferation.^[41]^ On the other hand, when the pathway is upregulated, tissue degeneration and inhibition of cell growth are observed, —a commonality to AD and FTD.^[41,42]^ Additionally, the apoptotic transcription factor FOXO3 is dysregulated in response to oligomeric β-amyloid as in AD.^[43]^ TDP-43 may also interfere with the FOXO pathway’s ability to regulate RNA metabolism and protein quality control in animal models.^[44]^ These pathways have an emerging role in neurodegenerative diseases and are potential candidates for therapeutics based on their association with differential expression of neuronal SEV miRNA.

Lastly, our study group only identified miRNA-21-5p as differentially expressed within neuron-derived L1CAM (+) SEV isolated from the plasma and CSF. These results are encouraging, as they demonstrate that neuron derived L1CAM (+) SEV content is highly correlated across body fluids. This replicates previous work that neuron-derived L1CAM (+) SEV pass readily through the blood-brain barrier and provides preliminary data that plasma is a viable biofluid for studying neuronal L1CAM (+) SEV.^[13,14]^

Our study is one of the few studies on SEV miRNAs in FTD. Schneider et al compared SEV miRNA isolated from the CSF in 38 FTD autosomal dominant variant carriers (22 *GRN*, 11 *C9orf72* and 5 *MAPT*), 11 first-degree relatives who tested negative for an FTD variant, 17 participants with sporadic FTD, 13 participants with AD, and 10 healthy controls of similar age.^[45]^ They found that SEV with subtypes of miRNA (miR-204-5p and miR-632) were significantly decreased in genetic forms of FTD, while in sporadic FTD only miR-632 was significantly decreased compared with AD and healthy aging.^[45]^ Additionally, miR-204-5p and miR-632 were significantly decreased in symptomatic compared with pre-symptomatic variant carriers.^[45]^ While CSF is the most proximal fluid to cerebral processes, and the invasive nature of lumbar punctures limits the study of SEV miRNA. Sproviero et al compared the SEV miRNA isolated from the plasma of nine FTD participants to SEV miRNA in 6 AD participants, 6 amyotrophic lateral sclerosis participants, and nine Parkinson’s disease participants.^[46]^ Sproviero et al analyzed the predicted functions of differentially expressed SEV miRNA and found that dysregulated SEV miRNA in FTD were involved in pathway release of cytochrome c from mitochondria, mitotic G1 DNA damage checkpoint and DNA damage response, and signal transduction by p53 class mediator, resulting in cell cycle arrest.^[46]^ Thus, previous studies of FTD have demonstrated unique SEV miRNA profiles compared to healthy aging and other neurodegenerative diseases, with specific metabolic pathways impacted. However, SEV in these studies were isolated based on size and were not enriched for cells of origin (e.g., neurons or microglia) and therefore may have reflected other tissues (including liver, kidneys, and muscle).

We acknowledge some important limitations of this study. This preliminary study included only 33 participants, which is not yet a large enough sample size to translate these findings into clinical use. Additionally, since the majority of our FTD participants did not have known genetic forms of the disease, where pathophysiology is better understood, we cannot draw conclusions about the relationship between L1CAM (+) SEV and underlying FTLD pathologic mechanisms. Lastly, our FTD group was significantly more cognitively impaired based on CDR disease severity than the AD group. Future studies should strive to match participants with neurodegenerative disease based on disease severity. Overall, our study provides important early data on the unique signature of neuronal SEV miRNA expression in FTD patients. This serves as the foundation for future research in larger studies examining neuronal SEV across neurodegenerative diseases, both for diagnostics and therapeutics.

## Author contributions

**Conceptualization:** Scott Langevin, Russell P. Sawyer.

**Data curation:** Jacek Biesiada, Damaris Kuhnell, Mario Medvedovic.

**Formal analysis:** Xiang Zhang, Jacek Biesiada, Mario Medvedovic, Scott Langevin.

**Funding acquisition:** Russell P. Sawyer.

**Investigation:** Damaris Kuhnell, Hannah L. Greenland, Scott Langevin, Russell P. Sawyer

**Methodology:** Xiang Zhang, Jacek Biesiada, Damaris Kuhnell, Hannah L. Greenland, Mario Medvedovic, Scott Langevin.

**Supervision:** Russell P. Sawyer.

**Writing – original draft:** Jonathan Pounders, Emily J. Hill, Russell P. Sawyer.

**Writing – review & editing:** Jonathan Pounders, Emily J. Hill, Destiny Hooper, Xiang Zhang, Leyla Esfandiari, Emerlee Timmerman, Forrest Foster, Chenran Wang, Kyle B. Walsh, Rhonna Shatz, Daniel Woo, Mario Medvedovic, Scott Langevin, Russell P. Sawyer.

## Supplementary Material


