# Supplementary material for: MicroRNA expression within neuronal-derived small extracellular vesicles in frontotemporal degeneration
Source: Medicine (Baltimore). 2022 Oct 7;101(40):e30854. doi: 10.1097/MD.0000000000030854 (PMC9542922; doi:10.1097/MD.0000000000030854)
Supplement: Supplementary file 1 [file medi-101-e30854-s001.pdf]

SUPPLEMENTARY TABLE #1 Complete List of MicroRNA Detected in Neuronal Small Extracellular Vesicles.

microRNA	Mean Control	Mean FTD	Fold Change	log^2 Fold Change	p-value
miRNA-181c	27.01	3.34	0.15	-2.72	<0.01
miRNA-3168	7.88	11.76	0.46	-1.12	0.08
miRNA-148a	47.88	25.23	0.56	-0.84	0.15
miRNA-22	2.04	6.38	2.63	1.40	0.18
miRNA-3545	7.41	19.07	2.35	1.23	0.19
miRNA-203	7.41	19.07	2.35	1.24	0.21
miRNA-122a	10.09	10.09	2.28	1.19	0.21
miRNA-3591	10.09	10.09	2.28	1.19	0.22
miRNA-126	14.20	26.82	1.53	0.61	0.35
miRNA-184	65.21	239.87	1.48	0.57	0.43
miRNA-10395	4.15	11.74	1.42	0.5	0.45
miRNA-151	9.62	8.57	0.84	-0.25	0.64
let-7c	12.90	19.42	1.36	0.44	0.69
miRNA-423	10.71	20.54	1.15	0.21	0.75
miRNA-320b	4.33	8.37	0.82	-0.29	0.75
miRNA-3184	10.71	20.54	1.16	0.21	0.75
miRNA-451	10.49	13.66	1.15	0.20	0.77
let-7b	10.32	14.18	1.11	0.15	0.83
miRNA-1	5.36	7.77	1.14	0.19	0.83
miRNA-21	54.56	62.01	0.95	-0.07	0.89
let-7i	12.89	16.44	1.03	0.04	0.96
let-7f	5.07	6.87	0.93	-0.11	0.99
let-7g	3.80	4.62	0.93	-0.11	0.99

FTD = frontotemporal dementia, miRNA = microRNA
