# Supplementary material for: MicroRNA expression within neuronal-derived small extracellular vesicles in frontotemporal degeneration
Source: Medicine (Baltimore). 2022 Oct 7;101(40):e30854. doi: 10.1097/MD.0000000000030854 (PMC9542922; doi:10.1097/MD.0000000000030854)
Supplement: Supplementary file 2 [file medi-101-e30854-s002.pdf]

SUPPLEMENTARY TABLE # 2 KEGG pathway in Frontotemporal Dementia vs Controls

KEGG pathway	p-value	# of genes	miRNAs
Prion diseases	1.05E-10	7	miR-22-3p<br>miR-148a-3p<br>miR-181c-3p
Fatty acid biosynthesis	1.33E-09	3	miR-148a-3p<br>miR-22-3p<br>miR-181c-3p
Hippo signaling pathway	1.33E-09	45	miR-22-3p<br>miR-3168<br>miR-148a-3p<br>miR-181c-3p<br>miR-203b-5p
Viral carcinogenesis	4.18E-07	68	miR-22-3p<br>miR-148a-3p<br>miR-181c-3p<br>miR-203b-5p
Proteoglycans in cancer	7.81E-06	61	miR-22-3p<br>miR-148a-3p<br>miR-181c-3p<br>miR-203b-5p
TGF-beta signaling pathway	2.29E-05	27	miR-22-3p<br>miR-3168<br>miR-148a-3p<br>miR-181c-3p
Renal cell carcinoma	2.29E-05	28	miR-22-3p<br>miR-3168<br>miR-148a-3p<br>miR-181c-3p<br>miR-203b-5p
Cell cycle	3.87E-05	48	miR-22-3p<br>miR-148a-3p<br>miR-181c-3p<br>miR-203b-5p
Transcriptional misregulation in cancer	4.05E-05	58	miR-22-3p<br>miR-3168<br>miR-148a-3p

			miR-181c-3p miR-203b-5p
Central carbon metabolism in cancer	4.15E-05	25	miR-22-3p miR-148a-3p miR-181c-3p miR-203b-5p
FoxO signaling pathway	0.000163	47	miR-22-3p miR-3168 miR-148a-3p miR-181c-3p miR-203b-5p
Hepatitis B	0.000757	43	miR-22-3p miR-148a-3p miR-181c-3p miR-203b-5p
Chronic myeloid leukemia	0.000965	28	miR-22-3p miR-148a-3p miR-181c-3p miR-203b-5p
Endocytosis	0.001101	66	miR-22-3p miR-3168 miR-148a-3p miR-181c-3p miR-203b-5p
Glycosaminoglycan biosynthesis - keratan sulfate	0.00133	6	miR-22-3p miR-148a-3p miR-181c-3p miR-203b-5p
Epstein-Barr virus infection	0.001505	63	miR-22-3p miR-3168 miR-148a-3p miR-181c-3p miR-203b-5p
Lysine degradation	0.002918	14	miR-22-3p miR-3168 miR-148a-3p miR-181c-3p miR-203b-5p
Steroid biosynthesis	0.004031	7	miR-22-3p miR-148a-3p
Signaling pathways regulating	0.005723	44	miR-22-3p miR-3168

pluripotency of stem cells			miR-148a-3p<br>miR-181c-3p<br>miR-203b-5p
Estrogen signaling pathway	0.005723	33	miR-22-3p<br>miR-3168<br>miR-148a-3p<br>miR-181c-3p<br>miR-203b-5p
Pancreatic cancer	0.006354	24	miR-22-3p<br>miR-3168<br>miR-148a-3p<br>miR-203b-5p
Colorectal cancer	0.00678	23	miR-22-3p<br>miR-3168<br>miR-148a-3p<br>miR-181c-3p<br>miR-203b-5p
Pathways in cancer	0.009918	111	miR-22-3p<br>miR-3168<br>miR-148a-3p<br>miR-181c-3p<br>miR-203b-5p
Gap junction	0.014516	33	miR-22-3p<br>miR-148a-3p<br>miR-181c-3p<br>miR-203b-5p
Glioma	0.014516	22	miR-22-3p<br>miR-3168<br>miR-148a-3p<br>miR-181c-3p<br>miR-203b-5p
Prostate cancer	0.017033	31	miR-22-3p<br>miR-3168<br>miR-148a-3p<br>miR-181c-3p<br>miR-203b-5p
Antigen processing and presentation	0.027344	22	miR-22-3p<br>miR-148a-3p<br>miR-181c-3p<br>miR-203b-5p
Ubiquitin mediated proteolysis	0.030559	43	miR-22-3p<br>miR-3168

			miR-148a-3p<br>miR-181c-3p
Adherens junction	0.032351	23	miR-22-3p<br>miR-148a-3p<br>miR-181c-3p
Endometrial cancer	0.032351	19	miR-22-3p<br>miR-3168<br>miR-148a-3p<br>miR-181c-3p<br>miR-203b-5p
Sulfur metabolism	0.032889	3	miR-148a-3p<br>miR-181c-3p
Protein processing in endoplasmic reticulum	0.033103	51	miR-22-3p<br>miR-3168<br>miR-148a-3p<br>miR-181c-3p<br>miR-203b-5p
Progesterone-mediated oocyte maturation	0.037928	29	miR-22-3p<br>miR-148a-3p<br>miR-181c-3p
Huntington's disease	0.049355	46	miR-22-3p<br>miR-3168<br>miR-148a-3p<br>miR-181c-3p<br>miR-203b-5p
