# Supplementary material for: MicroRNA expression within neuronal-derived small extracellular vesicles in frontotemporal degeneration
Source: Medicine (Baltimore). 2022 Oct 7;101(40):e30854. doi: 10.1097/MD.0000000000030854 (PMC9542922; doi:10.1097/MD.0000000000030854)
Supplement: Supplementary file 3 [file medi-101-e30854-s003.pdf]

SUPPLEMENTARY TABLE #3 KEGG Pathway Analysis of Alzheimer's Disease vs Healthy Controls

KEGG pathway	p-value	# of genes	#miRNAs
Proteoglycans in cancer	2.37E-10	90	miR-122-5p<br>miR-22-3p<br>miR-3591-3p<br>miR-148a-3p<br>miR-181c-3p<br>miR-203a-3p<br>miR-203b-5p
Hippo signaling pathway	2.69E-09	59	miR-122-5p<br>miR-22-3p<br>miR-3591-3p<br>miR-148a-3p<br>miR-181c-3p<br>miR-203a-3p<br>miR-203b-5p
Prion diseases	8.54E-08	9	miR-122-5p<br>miR-22-3p<br>miR-3591-3p<br>miR-148a-3p<br>miR-181c-3p
TGF-beta signaling pathway	2.32E-07	37	miR-122-5p<br>miR-22-3p<br>miR-3591-3p<br>miR-148a-3p<br>miR-181c-3p<br>miR-203a-3p
Fatty acid biosynthesis	8.67E-07	4	miR-122-5p<br>miR-22-3p<br>miR-148a-3p<br>miR-181c-3p
Chronic myeloid leukemia	1.28E-06	40	miR-122-5p<br>miR-22-3p<br>miR-3591-3p<br>miR-148a-3p<br>miR-181c-3p<br>miR-203a-3p<br>miR-203b-5p
Viral carcinogenesis	3.44E-06	79	miR-122-5p<br>miR-22-3p<br>miR-3591-3p

			miR-148a-3p<br>miR-181c-3p<br>miR-203b-5p
Gap junction	1.85E-05	43	miR-122-5p<br>miR-22-3p<br>miR-3591-3p<br>miR-148a-3p<br>miR-181c-3p<br>miR-203a-3p<br>miR-203b-5p
Glycosaminoglycan biosynthesis - keratan sulfate	2.32E-05	9	miR-122-5p<br>miR-22-3p<br>miR-3591-3p<br>miR-148a-3p<br>miR-181c-3p<br>miR-203b-5p
Renal cell carcinoma	3.55E-05	35	miR-122-5p<br>miR-22-3p<br>miR-3591-3p<br>miR-148a-3p<br>miR-181c-3p<br>miR-203a-3p<br>miR-203b-5p
Glioma	3.55E-05	32	miR-122-5p<br>miR-22-3p<br>miR-3591-3p<br>miR-148a-3p<br>miR-181c-3p<br>miR-203a-3p<br>miR-203b-5p
Prostate cancer	3.55E-05	45	miR-122-5p<br>miR-22-3p<br>miR-3591-3p<br>miR-148a-3p<br>miR-181c-3p<br>miR-203a-3p<br>miR-203b-5p
Cell cycle	3.76E-05	58	miR-122-5p<br>miR-22-3p<br>miR-3591-3p<br>miR-148a-3p<br>miR-181c-3p<br>miR-203a-3p

			miR-203b-5p
FoxO signaling pathway	6.11E-05	59	miR-122-5p<br>miR-22-3p<br>miR-3591-3p<br>miR-148a-3p<br>miR-181c-3p<br>miR-203a-3p<br>miR-203b-5p
Hepatitis B	6.11E-05	59	miR-122-5p<br>miR-22-3p<br>miR-3591-3p<br>miR-148a-3p<br>miR-181c-3p<br>miR-203a-3p<br>miR-203b-5p
Thyroid hormone signaling pathway	8.43E-05	53	miR-122-5p<br>miR-22-3p<br>miR-3591-3p<br>miR-148a-3p<br>miR-181c-3p<br>miR-203a-3p
Central carbon metabolism in cancer	0.000102	32	miR-122-5p<br>miR-22-3p<br>miR-3591-3p<br>miR-148a-3p<br>miR-181c-3p<br>miR-203b-5p
Other types of O-glycan biosynthesis	0.000153	13	miR-122-5p<br>miR-22-3p<br>miR-3591-3p<br>miR-148a-3p<br>miR-181c-3p
Insulin signaling pathway	0.00047	63	miR-122-5p<br>miR-22-3p<br>miR-3591-3p<br>miR-148a-3p<br>miR-181c-3p<br>miR-203a-3p<br>miR-203b-5p
Pathways in cancer	0.000584	149	miR-122-5p<br>miR-22-3p<br>miR-3591-3p<br>miR-148a-3p

			miR-181c-3p<br>miR-203a-3p<br>miR-203b-5p
Adherens junction	0.000592	33	miR-122-5p<br>miR-22-3p<br>miR-3591-3p<br>miR-148a-3p<br>miR-181c-3p<br>miR-203a-3p
Pancreatic cancer	0.000592	32	miR-122-5p<br>miR-22-3p<br>miR-3591-3p<br>miR-148a-3p<br>miR-203a-3p<br>miR-203b-5p
Transcriptional misregulation in cancer	0.000617	69	miR-122-5p<br>miR-22-3p<br>miR-3591-3p<br>miR-148a-3p<br>miR-181c-3p<br>miR-203a-3p<br>miR-203b-5p
Estrogen signaling pathway	0.000641	42	miR-122-5p<br>miR-22-3p<br>miR-3591-3p<br>miR-148a-3p<br>miR-181c-3p<br>miR-203b-5p
Signaling pathways regulating pluripotency of stem cells	0.001192	57	miR-122-5p<br>miR-22-3p<br>miR-3591-3p<br>miR-148a-3p<br>miR-181c-3p<br>miR-203a-3p<br>miR-203b-5p
Neurotrophin signaling pathway	0.001802	53	miR-122-5p<br>miR-22-3p<br>miR-3591-3p<br>miR-148a-3p<br>miR-181c-3p<br>miR-203a-3p<br>miR-203b-5p
mTOR signaling pathway	0.002913	29	miR-122-5p

			miR-22-3p<br>miR-3591-3p<br>miR-148a-3p<br>miR-181c-3p<br>miR-203a-3p<br>miR-203b-5p
Non-small cell lung cancer	0.007094	26	miR-122-5p<br>miR-22-3p<br>miR-3591-3p<br>miR-148a-3p<br>miR-203a-3p
Epstein-Barr virus infection	0.007789	77	miR-122-5p<br>miR-22-3p<br>miR-3591-3p<br>miR-148a-3p<br>miR-181c-3p<br>miR-203a-3p<br>miR-203b-5p
Small cell lung cancer	0.007981	39	miR-122-5p<br>miR-22-3p<br>miR-3591-3p<br>miR-148a-3p<br>miR-181c-3p<br>miR-203a-3p<br>miR-203b-5p
Glycosylphosphatidylinositol(GPI)-anchor biosynthesis	0.00823	13	miR-122-5p<br>miR-22-3p<br>miR-3591-3p<br>miR-148a-3p<br>miR-181c-3p
Endocytosis	0.00934	76	miR-122-5p<br>miR-22-3p<br>miR-3591-3p<br>miR-148a-3p<br>miR-181c-3p<br>miR-203a-3p<br>miR-203b-5p
Focal adhesion	0.00934	80	miR-122-5p<br>miR-22-3p<br>miR-3591-3p<br>miR-148a-3p<br>miR-181c-3p<br>miR-203a-3p

			miR-203b-5p
Endometrial cancer	0.011328	25	miR-122-5p<br>miR-22-3p<br>miR-3591-3p<br>miR-148a-3p<br>miR-181c-3p<br>miR-203a-3p<br>miR-203b-5p
2-Oxocarboxylic acid metabolism	0.014662	7	miR-122-5p<br>miR-22-3p<br>miR-3591-3p
Ubiquitin mediated proteolysis	0.014662	56	miR-122-5p<br>miR-22-3p<br>miR-3591-3p<br>miR-148a-3p<br>miR-181c-3p<br>miR-203a-3p
AMPK signaling pathway	0.014662	50	miR-122-5p<br>miR-22-3p<br>miR-3591-3p<br>miR-148a-3p<br>miR-181c-3p<br>miR-203a-3p<br>miR-203b-5p
Sulfur metabolism	0.015062	4	miR-122-5p<br>miR-148a-3p<br>miR-181c-3p<br>miR-203a-3p
HIF-1 signaling pathway	0.016506	44	miR-122-5p<br>miR-22-3p<br>miR-3591-3p<br>miR-148a-3p<br>miR-181c-3p<br>miR-203a-3p
Rap1 signaling pathway	0.016506	77	miR-122-5p<br>miR-22-3p<br>miR-3591-3p<br>miR-148a-3p<br>miR-181c-3p<br>miR-203a-3p<br>miR-203b-5p
Melanoma	0.016506	31	miR-122-5p<br>miR-22-3p

			miR-3591-3p<br>miR-148a-3p<br>miR-181c-3p<br>miR-203a-3p<br>miR-203b-5p
Dorso-ventral axis formation	0.019066	15	miR-122-5p<br>miR-22-3p<br>miR-3591-3p<br>miR-148a-3p
Colorectal cancer	0.023831	27	miR-122-5p<br>miR-22-3p<br>miR-148a-3p<br>miR-181c-3p<br>miR-203b-5p
MAPK signaling pathway	0.025619	92	miR-122-5p<br>miR-22-3p<br>miR-3591-3p<br>miR-148a-3p<br>miR-181c-3p<br>miR-203a-3p<br>miR-203b-5p
ErbB signaling pathway	0.035829	35	miR-122-5p<br>miR-22-3p<br>miR-3591-3p<br>miR-148a-3p<br>miR-181c-3p<br>miR-203a-3p<br>miR-203b-5p
Regulation of actin cytoskeleton	0.035829	77	miR-122-5p<br>miR-22-3p<br>miR-3591-3p<br>miR-148a-3p<br>miR-181c-3p<br>miR-203a-3p<br>miR-203b-5p
Bacterial invasion of epithelial cells	0.035829	30	miR-122-5p<br>miR-22-3p<br>miR-3591-3p<br>miR-148a-3p<br>miR-181c-3p<br>miR-203a-3p<br>miR-203b-5p

Progesterone-mediated oocyte maturation	0.040168	36	miR-122-5p<br>miR-22-3p<br>miR-3591-3p<br>miR-148a-3p<br>miR-181c-3p
PI3K-Akt signaling pathway	0.043069	119	miR-122-5p<br>miR-22-3p<br>miR-3591-3p<br>miR-148a-3p<br>miR-181c-3p<br>miR-203a-3p<br>miR-203b-5p
Valine, leucine and isoleucine biosynthesis	0.048163	2	miR-22-3p<br>miR-3591-3p
