# Supplementary material for: MicroRNA expression within neuronal-derived small extracellular vesicles in frontotemporal degeneration
Source: Medicine (Baltimore). 2022 Oct 7;101(40):e30854. doi: 10.1097/MD.0000000000030854 (PMC9542922; doi:10.1097/MD.0000000000030854)
Supplement: Supplementary file 4 [file medi-101-e30854-s004.pdf]

SUPPLEMENTARY TABLE #4 Comparison of Cerebral Spinal Fluid and Plasma Concentrations of Neuronal Small Extracellular Vesicle MicroRNA

MicroRNA	Fold Change	log^2 Fold Change	p-value
Let-7b-5p	1.09	0.13	0.90
miRNA-21-5p	4.48	2.16	0.10
miRNA-148a-3p	0.48	-1.07	0.31
miRNA-184	1.11	0.15	0.87
miRNA-181c	0.79	-0.34	0.79
